# Antioxidant properties of Milk and dairy products: a comprehensive review of the current knowledge

**DOI:** 10.1186/s12944-019-0969-8

**Published:** 2019-02-04

**Authors:** Imran Taj Khan, Muhammad Nadeem, Muhammad Imran, Rahman Ullah, Muhammad Ajmal, Muhammad Hayat Jaspal

**Affiliations:** 1 Department of Dairy Technology, University of Veterinary and Animal Sciences, Lahore, Punjab Pakistan; 2 Institute of Home and Food Sciences, Faculty of Life Sciences, Government College University, Faisalabad, Punjab Pakistan; 3 Department of Meat Science and Technology, University of Veterinary and Animal Sciences, Lahore, Punjab Pakistan

**Keywords:** Antioxidants activity, Milk, Carotenoids, Enzymatic antioxidants, Phytochemicals

## Abstract

Milk and dairy products are integral part of human nutrition and they are considered as the carriers of higher biological value proteins, calcium, essential fatty acids, amino acids, fat, water soluble vitamins and several bioactive compounds that are highly significant for several biochemical and physiological functions. In recent years, foods containing natural antioxidants are becoming popular all over the world as antioxidants can neutralize and scavenge the free radicals and their harmful effects, which are continuously produced in the biological body. Uncontrolled free radicals activity can lead to oxidative stresses, which have been implicated in breakdown of vital biochemical compounds such as lipids, protein, DNA which may lead to diabetes, accelerated ageing, carcinogenesis and cardiovascular diseases. Antioxidant capacity of milk and milk products is mainly due to sulfur containing amino acids, such as cysteine, phosphate, vitamins A, E, carotenoids, zinc, selenium, enzyme systems, superoxide dismutase, catalase, glutathione peroxidase, milk oligosaccharides and peptides that are produced during fermentation and cheese ripening. Antioxidant activity of milk and dairy products can be enhanced by phytochemicals supplementation while fermented dairy products have been reported contained higher antioxidant capacity as compared to the non-fermented dairy products. Literature review has shown that milk and dairy products have antioxidant capacity, however, information regarding the antioxidant capacity of milk and dairy products has not been previously compiled. This review briefly describes the nutritional and antioxidant capacity of milk and dairy products.

## Background

**Fig. 1:**
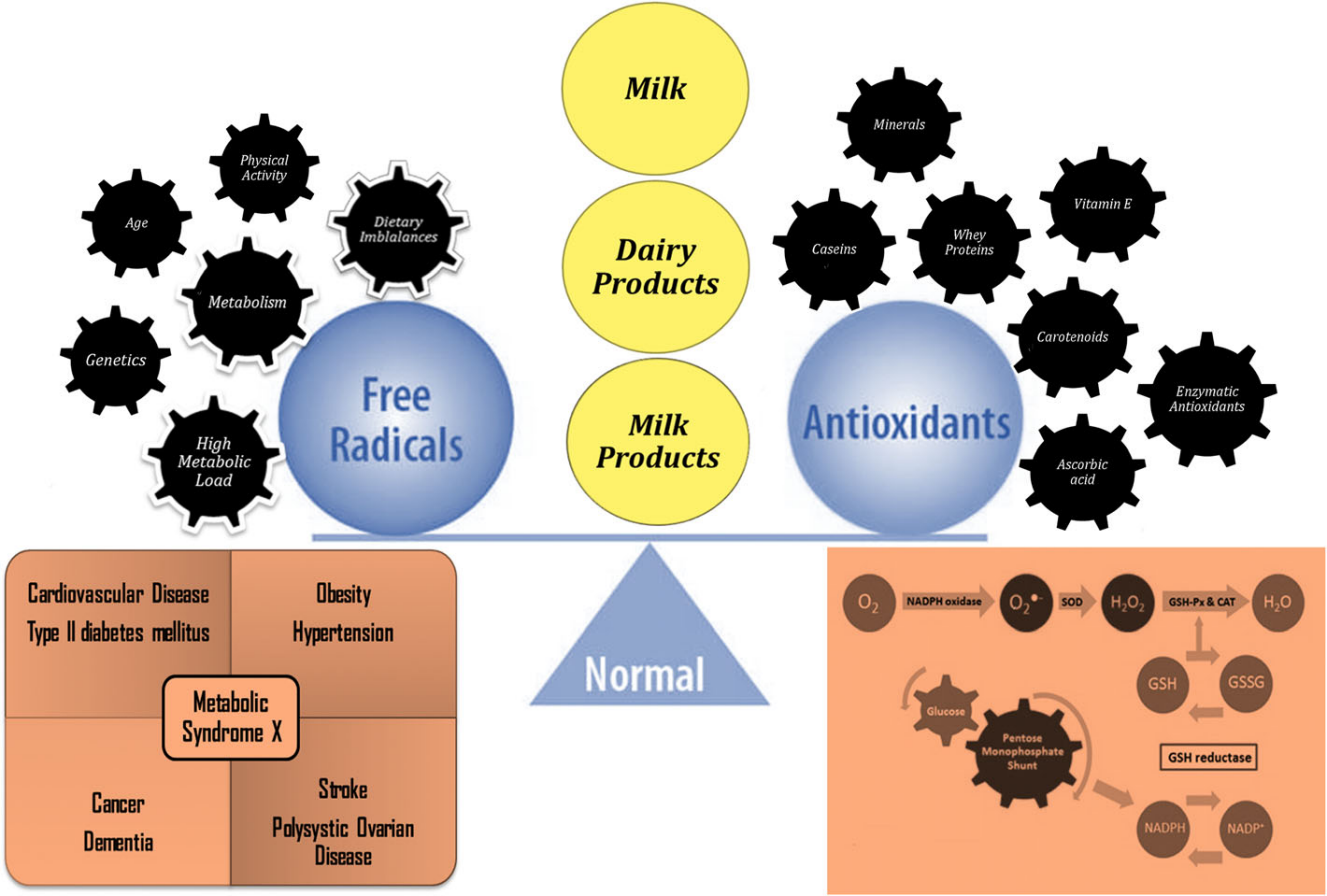
Representative figure for antioxidant properties of milk and dairy products

Dairy products constitute about 25–30% of the average diet of an individual [1] (Fig. 1). Milk and milk products are nutritious food items containing numerous essential nutrients such as, oleic acid, conjugated linoleic acid, omega-3 fatty acids, vitamins, minerals and bioactive compounds such as antioxidants [2]. Antioxidants are chemical substances that can neutralize and scavenge the free radicals, which are continuously produced in the body [3]. For the generation of energy, oxidation is indispensable to living organism for biological processes. However, oxidative stress can cause serious damage to biological systems. It is scientifically established that reactive oxygen species are unceasingly produced in human body. Uncontrolled free radicals in body can lead to oxidative stresses, which may consequence in atherosclerosis, diabetes, accelerated ageing, cardiovascular diseases and break down of vital biochemical compounds [4]. Intake of antioxidants in the form of antioxidative supplements of foods rich in antioxidants may protect the body from oxidative stress and damage [5]. Metabolic diseases are closely correlated with life style and changes in life style has a great deal of impact on disease patterns, about 20–30 years ago, infectious disease were more than non-communicable diseases, but now the non-communicable/metabolic diseases are on the higher side. In current scenario, healthy/functional foods should be selected to avoid or minimize non-communicable diseases, such as diabetes, cancers and cardiovascular diseases [6]. Demand for foods containing natural antioxidants is increasing across the globe. Large number of foods and dairy products are being supplemented with natural antioxidants [7]. Antioxidant capacity of milk and dairy products is due to sulfur containing amino acids cysteine, vitamins A, E, carotenoids, enzyme systems, superoxide dismutase, catalase and glutathione peroxidase [8]. Milk also contains appreciable amounts of equol, a polyphenolic metabolite of daidzein, antioxidant activity of this equol is scientifically established [9]. Superoxide radicals, hydroxyl radicals, and peroxide radicals can be inhibited by the antioxidant systems of milk [10]. Human body has mechanisms for the neutralization and scavenging of reactive oxygen species. Significant line of defense against reactive oxygen species are comprised of enzymes such as, glutathione peroxide, catalase and superoxide dismutase, ubiquinol and uric acid [11]. Lipid oxidation is the main reason for the chemical spoilage of food and dairy products and it leads to the production of objectionable changes in nutritional value, flavor and texture of foods [12]. Literature review has shown that milk and dairy products have antioxidant capacity, however, information regarding the antioxidant capacity of milk and dairy products has not been previously compiled. This paper briefly describes the antioxidant capacity of milk and dairy products.

## Antioxidant properties of caseins

Caseins are the major protein of bovine and ovine milks present in the form of macro-molecular aggregates. Due to the difference in phosphate content, various casein fractions are present in milk, for example, phosphate content of α, β and κ caseins are 10, 5 and 1 mol per casein mole and phosphate can provide antioxidant activity to the casein micelles [13]. Milk proteins have shown antioxidant activity for the scavenging of reactive oxygen species. Studies have shown that casein inhibited the lipoxygenase-catalyzed lipid autoxidation. Free amino acids cannot quench the free radicals and for the scavenging of free radicals, primary structure of casein molecules acts as scavenger [14]. Phosphoserine residues associated with casein molecules and inorganic phosphate present in casein and serum can bind the non-heme iron. In a previous investigation, it was observed that 72 and 21% of the supplemented non-heme iron in skim milk was recovered from αs- and β-caseins and phosphoseryl rich peptides of casein phosphatides can bind the divalent metal and casein derived peptides inhibited lipoxygenase activity [15]. Casein derived phosphopeptides revealed the capability of sequestering iron in lipid and aqueous food systems [16]. Browning is a serious problem in many foods and casein based coatings are commercially used to prevent oxidation induced browning of fruits and vegetables. Efficacy of calcium caseinate and whey powder in delaying the enzymatic browning in sliced potatoes and apples were investigated and results showed that milk protein based edible coating efficiently postponed the enzymatic browning. Whey protein powder revealed better antioxidant activity than calcium caseinate and the differences in antioxidant activity of whey protein and caseinate was attributed to the difference in amino acid profile [17]. Antioxidant activities of superoxide dismutase, catalase and glutathione peroxidase, casein and certain peptides are well established [18].

## Antioxidant properties of whey proteins

In recent years, utilization of whey in food and nonfood applications is mounting across the globe. Whey protein has higher biological value and despite the fact that about 30–35% of the whey is still discarded [19]. In food industries, whey proteins are used as emulsifying, gelling and bulking agent. Antioxidant activity of whey protein is scientifically established and antioxidants of whey can efficiently inhibit the lipid oxidation [20]. Antioxidant activity of whey protein is due to the chelation of transition metals by lactoferrin and scavenging of free radicals by sulphur containing amino acids [21]. Whey proteins boost the level of glutathione peroxidase which is regarded as one of the most significant water soluble antioxidant system [22]. Whey proteins have antioxidant activity and addition of whey protein in soybean oil emulsion increased the oxidative stability [12]. Antioxidant characteristics of salmon oil emulsion increased as a function of addition of whey protein [20]. Food containing whey proteins have better antioxidant activity. Lactoferrin and casein can inhibit lipid peroxidation, generation of peroxide radicals, thiobarbituric acid reacting substances, uptake of oxygen and iron oxide free radicals [23]. Casein fraction, composition of whey proteins and amino acids profile of cow, buffalo, sheep and goat milk have been presented in Tables 1, 2 and 3, respectively.

**Table 1:** Casein fraction of cow, buffalo, goat and sheep milk

Parameters	Cow	Buffalo	Sheep	Goat
TPC (g/L)	27.8	49.2	59.4	33.4
αS_1_-casein (%)	37	90	33	99
αS_2_-casein (%)	7	13	14	8.52
β-casein (%)	42	28	30	63
γ-casein (%)	6	22	9	18
κ- casein (%)	9	7	14	8

*TPC* Total Protein Content

Source of Data

Cow Milk: [104]

Buffalo Milk: [105]

Sheep Milk: [104]

Goat Milk: [104]

**Table 2:** Composition of whey proteins in cow, buffalo, goat and sheep milk

Parameters	Cow Milk	Buffalo Milk	Sheep Milk	Goat Milk
Whey proteins (g/L)	6.46	6.46	10.76	6.14
β- Lactoglobulin (%)	59.3	59.3	61.1	54.2
α- lactalbumin (%)	16.2	16.2	10.8	21.4
Immunoglobulin’s (%)	15.0	15.0	20.0	11.5
Serum albumin/lactoferrin (%)	9.5	9.5	8.1	12.8

Source of Data

Cow Milk: [104]

Buffalo Milk: [105]

Sheep Milk: [104]

Goat Milk: [105]

**Table 3:** Amino acids profile of cow, buffalo, sheep and goat milk

Amino Acid (g/100 g)	Cow Milk	Goat Milk	Buffalo Milk	Sheep Milk
Aspartic acid	7.8	7.4	7.13	6.5
Threonine	4.5	5.7	5.714	4.4
Serine	4.8	5.2	4.65	3.4
^a^Glutamic acid	23.2	19.3	21.4	14.5
Proline	9.6	14.6	12.0	16.2
^a^Cystine	0.6	0.6	0.586	0.9
Glycine	1.8	2.1	1.93	3.5
Alanine	3.0	3.6	3.03	2.4
^a^Valine	4.8	5.7	6.760	6.4
^a^Methionine	1.8	3.5	0.928	2.7
^a^Isoleucine	4.2	7.1	5.714	4.6
^a^Leucine	8.7	8.2	9.792	9.9
^a^Tyrosine	4.5	4.8	3.858	3.8
Phenylalanine	4.8	6.0	4.713	4.3
^a^Histidine	3.0	5.0	2.73	6.7
Lysine	8.1	8.2	7.497	7.8

^a^Amino acid has antioxidant activity in milk and dairy products

Source of Data

Cow Milk: [106]

Goat Milk: [107]

Buffalo Milk: [108]

Sheep Milk: [107]

## Antioxidant characteristics of carotenoids

Carotenoids are lipophilic molecules with a tendency to accrue in membrane or lipoproteins [24]. Milk fat globule membrane is considered as the most volatile site for auto-oxidation [24]. β-carotene is regarded as preventive antioxidant, it can quench singlet oxygen and one molecule of β-carotene can quench 250 to 1000 molecules of singlet oxygen [26]. Carotenoids act as scavengers of singlet oxygen and other reactive oxygen species [25]. Among the various antioxidant systems in milk, carotenoids act a scavenger of singlet oxygen and peroxyl radicals [27]. Dairy lipids may suffer from oxidation, which leads to the negative impact on quality and sensory characteristics of finished products. Auto-oxidation and light induced oxidation is affected by a complex interaction of pro and antioxidants. Photo-oxidation is predominantly inhibited by β-carotene, it absorbs light that would otherwise be absorbed by riboflavin, which may give rise to quality related issues. β-carotene absorbs light in a concentration dependent manner [28]. Results of an earlier investigation regarding the migration of carotenoids from milk to cheese and butter have shown that concentration of carotenoids was intensified in cheese and butter [29].

## Antioxidant characteristics of ascorbic acid, vitamin E and minerals

Nutraceuticals and functional food ingredients that are beneficial to vascular health may represent useful compounds that are able to reduce the overall cardiovascular risks [30]. Ascorbic acid is one of the most strong and least toxic natural antioxidant. It is the main water soluble antioxidant present in milk and free radical scavenging activity of ascorbic acid is due to low oxidation-reduction potential (330 mV). Ascorbic acid is the major water-soluble antioxidant in milk and can act as strong free radical scavenger [31]. Ascorbic acid can scavenge superoxide anion radicals, alkoxyl radicals and singlet oxygen [31]. Ascorbic acid can scavenge superoxide, iron oxide, nitric oxide and alkoxyl radicals [32]. Ascorbic acid significantly inhibited the degradation of riboflavin in cream in presence of 1000 Lux light for four days [33]. Ascorbic acid and tocopherol were added in milk to enhance the flavor and photo-oxidative stability. Ascorbic acid and tocopherol supplemented samples revealed better flavor and photo-oxidative stability as compared to non-supplemented samples [34]. Ascorbic acid significantly inhibited the degradation of riboflavin in light exposed milk, antioxidant activities were mainly attributed to the scavenging effect on singlet oxygen [35]. A study was conducted to determine the effect of tocopherol and vitamin C against the development of atopy in infants. Increased concentration of vitamin C in breast milk reduced the risk of atopy in infants [36]. Ascorbic acid is extremely helpful for the infants as it plays a pivotal role in the formation of neuro transmitters, synthesis of carnitine and improves the absorption of iron. Human and cow milk contains about 40 and 20 mg/Liter [37, 38]. Oxidation of ascorbic acid depends upon temperature, light, oxygen and amount of catalysts. Vitamin A and E are regarded as primary lipid soluble antioxidants and main job of these vitamins is to protect the polyunsaturated fatty acids and associated bio-chemical compounds from peroxidation (Table 4). α-tocopherol can be considered one of the most important lipid-soluble antioxidants in milk, due to it is presence in milk fat globule membrane [39]. It can act as a preventative, chain breaking antioxidant and quencher of singlet oxygen in milk [40]. Milk can develop off flavor as a result of photo-oxidation and contamination with copper. The existence of antioxidants in milk can inhibit the free radical mechanism by donating the proton and thus inhibit the onset of auto-oxidation. Vitamin E can inhibit the activity of plasmin; a proteolytic enzyme and secondly it can directly scavenge the free radicals [41]. Among the tocopherols, α-tocopherol is regarded as more powerful scavenger of free radicals and antioxidant activity of β-, γ- and δ-tocopherol is about 80–90% less than α-tocopherol [42]. γ-tocopherol is of high functional value as it can trap the nitrogen oxide species. It helps the body to prevent cardiovascular diseases and cancers. The concentration of vitamin E in cow milk has been reported about 0.9 mg/mL while summer milk possessed higher concentration than winter milk. Concentration of vitamin in human milk ranges from 3 to 13 mg/mL [43]. Addition of 100 mg α-tocopherol/kg milk fat and 100 mg ascorbyl palmitate/kg milk fat to UHT milk decreased the concentration of hexanal during the storage period of 4 weeks [34]. Khan et al. [44] studied the effect of vitamin E supplementation on oxidative stability of sheep butter and supplementation of sheep butter with 60 mg/kg efficiently inhibited the lipid peroxidation and raised the shelf stability. Antioxidant activity of zinc and selenium for the inhibition of superoxide dismutase is scientifically proven [45]. Glutathione and selenium enhanced the functional value and antioxidant capacity of milk [46]. Mineral content of cow, buffalo, goat and sheep milk have been presented in Table 5.

**Table 4:** Vitamin content of cow, buffalo, goat and sheep milk

Vitamins	Cow Milk (mg/100 g)	Buffalo Milk (mg/100 g)	Goat Milk (mg/100 g)	Sheep Milk (mg/100 g)
Vitamin A^a^	46	69	185	146
Vitamin E^a^	0.21	0.19	0.03	–
Thiamine	0.05	0.05	0.068	0.08
Riboflavin	0.17	0.11	0.21	0.37
Niacin	0.09	0.17	0.27	0.416
Pantothenic acid	0.37	0.15	0.31	0.408
Vitamin B_6_	0.04	0.33	0.046	0.08
Vitamin B_12_	0.45	0.40	0.665	0.712
Biotin	2.0	13	1.5	0.93
Vitamin C^a^	0.09	2.5	1.29	4.16
Vitamin D	2.0	2.0	1.33	1.18

^a^Vitamin possesses antioxidant activity

Source of Data

Cow Milk: [37]

Buffalo Milk: [107]

Goat Milk: [108]

Sheep Milk: [107]

**Table 5:** Mineral content of cow, buffalo, goat and sheep milk

Minerals	Cow Milk	Buffalo Milk	Goat Milk	Sheep Milk
Calcium	122 (mg/100 g)	112 (mg/100 g)	134 (mg/100 g)	195–200 (mg/100 g)
Phosphorus^a^	119 (mg/100 g)	99 (mg/100 g)	121 (mg/100 g)	124–158 (mg/100 g)
Potassium	152 (mg/100 g)	92 (mg/100 g)	181 (mg/100 g)	136–140 (mg/100 g)
Magnesium	12 (mg/100 g)	8 (mg/100 g)	16 (mg/100 g)	18–21 (mg/100 g)
Sodium	58 (mg/100 g)	35 (mg/100 g)	41 (mg/100 g)	44–58 (mg/100 g)
Zinc^a^	530 (μg/100 g)	410 (μg/100 g)	56 (μg/100 g)	520–747 (μg/100 g)
Iron^b^	80 (μg/100 g)	161 (μg/100 g)	7.22 (μg/100 g)	72–122 (μg/100 g)
Copper^b^	60.58 (μg/100 g)	35 (μg/100 g)	5.13 (μg/100 g)	40–68 (μg/100 g)
Manganese	20 (μg/100 g)	27 (μg/100 g)	3.2 (μg/100 g)	5.39 (μg/100 g)
Iodine	2.1 (μg/100 g)	4 (μg/100 g)	2.2 (μg/100 g)	10.41 (μg/100 g)
Selenium^a^	0.96 (μg/100 g)	6 (μg/100 g)	1.33 (μg/100 g)	3.14 (μg/100 g)

^a^Chemical constituents has antioxidant activity in milk

^b^Chemical constituent has pro-oxidant activity

Source of Data

Cow Milk: [46]

Buffalo Milk: [107]

Goat Milk: [109]

Sheep Milk: [107]

## Enzymatic antioxidants

### Superoxide dismutase

Superoxide dismutase (SOD) catalyzes the removal of superoxide free radicals (O_2_^−^) and safeguards the cells from harmful effects by the following reaction.

$$ {20}_{2^{\hbox{-} }}+2\mathrm{H}\to {\mathrm{H}}_2{\mathrm{O}}_2+{\mathrm{O}}_2 $$

Catalase, glutathione peroxidase or other reducing agents converts H_2_O_2_ to H_2_O, hydrogen peroxide formed from O_2_^−^ and oxidases is eliminated by catalases and peroxidases [47]. Cytosolic Cu/Zn-SOD, mitochondrial Mn-SOD and extracellular EC-SOD are the major forms of SOD [48]. SOD can inhibit lipid peroxidation. In cow milk SOD is exclusively present in skim milk fraction, with a concentration of 0.15 mg to 2.4 mg/L [49]. Human milk has 2.0 to 2.3 time higher concentration of SOD than cow milk.

## Glutathione peroxidase (GSHPx)

GSHPx is a selenium encompassing enzyme that provides protection against lipid peroxidation. It catalysis the breakdown of H_2_O_2_ and organic hydroperoxides (R-OOH) by glutathione (γGlu.Cys.Gly) as per following chemical reaction [50].

$$ \mathrm{ROOH}+2\mathrm{GSH}\to \mathrm{ROH}+\mathrm{GSSG}+\mathrm{H}2\mathrm{O} $$

More than 90% of GSHPx exists in milk as extra cellular enzyme and it is only enzyme which fixes selenium (about 30% of the total. Its concentration varies among the mammals and concentration is in the order of human > caprine > bovine [51]. Concentration of GSHPx in cow milk ranges from 12 to 30 U/mL and its activity is mainly dependent upon the concentration of selenium. Antioxidant activity and selenium content decreases with the progression of lactation [52].

## Catalase

Milk catalase is a heme protein and molecular weight of catalase is 200 kDa with isoelectric pH of 5.5. This enzyme is stable in a wide range of pH 5–10 and however, it rapidly loses activity out this pH range [53]. Most of the catalases contain heme and catalase causes the dismutation of H_2_O_2_ (a chemical reaction in which H_2_O_2_ causes oxidation of the other H_2_O_2_ molecules, consequently, one is converted to O_2_ and the other two are converted to tow molecules of H_2_O) [54]. A polarographic method showed that average catalase activity in cow milk was 1.95 U/mL [55]. Concentration of catalase in human milk is approximately ten times greater than cow milk [56].

## Oxidative stability of milk and milk products

The oxidative stability of milk and dairy products is of concern to the dairy industry. Oxidation in milk can result in strong off-flavors and in deterioration of the nutritional quality of milk. The oxidative stability of milk and dairy products is the result of a delicate balance between the anti- and pro-oxidative processes in milk. Oxidative stability of milk and dairy products depends upon fatty acid composition (Tables 6 and 7), contamination with metal ions, concentration of tocopherols and carotenoids [57]. Processing, packaging, storage conditions and period have a pronounced effect on the extent of natural antioxidants, which is directly connected with oxidative stability of pasteurized milk and dairy products [58]. It is extremely important to determine the antioxidant capacity of milk and milk products, as oxidation can only occur in case of an imbalance between the presence of reactive oxidants and the antioxidant defense mechanism [59]. Sensitivity to oxidation can also be monitored by measuring the antioxidative capacity of a product.

**Table 6:** Fatty acid profile of cow, buffalo, goat and sheep milk

Fatty acid	Cow Milk (g/100 g)	Buffalo Milk (g/100 g)	Goat Milk (g/100 g)	Sheep Milk (g/100 g)
C_4:0_	3.5	3.90	2.46	4.06
C_6:0_	2.3	2.33	2.40	2.78
C_8:0_	1.2	2.41	2.53	3.13
C_10:0_	2.6	2.40	9.38	4.97
C_12:0_	2.7	3.09	4.45	3.35
C_14:0_	9.3	10.64	10.16	10.16
C_16:0_	25.9	28.02	24.20	23.11
C_18:0_	14.3	12.58	12.51	12.88
^a^C_18:1_	27.6	24.10	23.01	26.01
^a^C_18:2_	2.1	2.04	2.72	1.61
^a^C_18:3_	0.7	0.68	0.53	0.92

^a^Fatty acids have a great impact on oxidative stability of milk and dairy products

Source of Data

Cow Milk: [56]

Buffalo Milk: [110]

Goat Milk: [106]

Sheep Milk: [111]

**Table 7:** Relative rates (M^− 1^ S^− 1^) of oxidation by triplet (autoxidation) and singlet (photo-oxidation) oxygen

Fatty acid	Triplet O_3_	Singlet O_2_
Oleic acid	1	3 × 10^4^
Linoleic acid	27	4 × 10^4^
Linolenic acid	77	7 × 10^4^

Source of Data

Triplet O_3_: [112]

Singlet O_2_: [112]

## Measuring antioxidant capacity and oxidative stability

Antioxidant capacity assays are useful in measuring the overall antioxidant activity in foods. Antioxidant capacity assays can be categorized into hydrogen atom transfer based assays and electron transfer based assays [33]. Zulueta et al. [60] reported that hydrogen atom transfer based assays measured antioxidant activity from amino acids in milk that can act as hydrogen donors. Determination of nitric oxide free radicals, total phenolic contents, flavonoid contents, DPPH free radicals, inhibition of oxidation of linolenic acid and total reducing capacity can be used for the characterization of antioxidant capacity in milk and dairy products [61]. Lipid oxidation in milk can be measured by several methods which include instrumental methods, such as transition in fatty acid profile, concentration of vitamin A, E and C and total antioxidant assays. Peroxide value measures the primary stages of auto-oxidation and it a useful parameter to determine the oxidation status of milk, cheese, butter and ice cream [28, 62]. Thiobarbituric acid test (TBA) has been used for the determination of secondary oxidation products [63]. Lim et al. [64] used gas chromatography for the determination of oxidation status of ice cream. Sensory techniques are also commonly used for the assessment of oxidized flavor in milk and milk products [65]. Antioxidant characteristics of some dairy products have been illustrated in Table 8.

**Table 8:** Antioxidant characteristics of some dairy products

Study design	Conclusions	Reference
Effect of grazing on antioxidant characteristics of sheep milk was investigated	Grazing improved the total antioxidant capacity of sheep milk	[65]
*Zingiber officinale* and *Beta vulgaris* were added in yoghurt milk to improve the antioxidant capacity of herbal yoghurt of buffalo, cow and goat milk yoghurt	Supplementation of yoghurt milk with *Zingiber officinale* and beta *vulgaris* improved the 2,2 diphenyl-1 picrylhydrazyl and ferric reducing antioxidant power in yoghurts	[113]
2,2 diphenyl-1 picrylhydrazyl and ferric reducing antioxidant power assays were used to determine the antioxidant capacity of milk along with conventional methods such as peroxide value, thiobarbituric acid value, loss of vitamins A & E	2,2 diphenyl-1 picrylhydrazyl and ferric reducing antioxidant power assays provided useful information regarding antioxidant capacity of milk	[72]
A study was to analyze the antioxidant capacity of yoghurts, acidophillus milks, butter milk and vegetable flavored fermented milk were analyzed for their antioxidant potential	Yoghurt and kefir were characterized by the highest antioxidant activity. The presence of probiotic *Lactobacillus casei* strains in the product positively improved the ferric reducing antioxidant power.	[114]
A study was conducted to estimate the effect of cow feed supplementation by carrots on the βcarotene and α-tocopherol concentration in butter oil	At the same time it contributed in more stable β-carotene, as well as 30% higher α-tocopherol concentration (*P* < 0.05)	[115]
A study was undertaken to assess the effect of betel leaves (*Piper betel Linn*) extract on the physico-chemical, sensory and antioxidant properties of khoa made from cow milk and stored under room temperature	Free fatty acids levels were well within the prescribed limit because of antioxidant properties exhibited by the aqueous extract of betel leaves. From the study, it was concluded that khoa with 0.5 aqueous extract of betel leaves restricted the production of free fatty acid compared to control due to antioxidant property of betel leaves	[116]
the antioxidant properties of kefir produced from goat milk with kefir grains were investigated using total phenolic contents,,2-Diphenyl-1-picrylhydrazyl assays	Antioxidant capacity of kefir was more than parent milk.	[117]
Antioxidant properties of milk oligosaccharides from various ruminants were studied	The result suggests that milk oligosaccharides derived from certain ruminant species could be used as natural antioxidants and further studies can be done to elucidate the role of milk oligosaccharides as a functional food and potential drug	[118]
The effect of *Pediococcus pentosaceus* on antioxidant characteristics of probiotic yoghurt was studied in cow, goat and camel milk	Results evidence that antioxidant of goat milk yoghurt was 93% as compared to 86 in camel milk. These results suggested that antioxidant characteristics of yogurt can be enhanced by probiotic bacteria	[119]
Cow milk was fermented by *Lactobacillus lactis* and *Lactobacillus delbeurkii*	Antioxidant capacity of milk fermented with *Lactobacillus, Lactobacillus* lactis and Lactobacillus delbeurkii were 21.91 and 29.7%	[120]
A study examined the effect of fish oil, Opal linseed and Szafir linseed on the antioxidants of Polish Holstein Friesian cow’s milk	The highest level of α-tocopherol was found in fish oil + Opal linseed group at the 21st day of supplementation. Total antioxidative status increased in all experimental groups; however, the highest peak was recorded in fish oil + Szafir linseed and Szafir linseed group	[121]
*Impact of Lactobacillus delbrueckii sp.bulgaricus, Lactobacillus rhamnosus, Streptococcus thermophilus* or *Lactobacillus delbrueckii* and *Lactobacillus fermentum* on antioxidant capacity of bovine milk and whey were investigated	Bacterial strains improved the DPPH free radical scavenging activity, Inhibition of superoxide anions, lipid oxidation and reduces the atherogenesis in humans	[122]
Effect of supplementation of Pirotski Kachkaval by ethanolic extract of *Kitaibelia vitifolia* on antioxidant characteristics were investigated	supplementation of Pirotski Kachkaval cheese by ethanolic extract of *Kitaibelia vitifolia* raised the antioxidant capacity of cheese	[123]
Antioxidant characteristics of ice cream was increased by partially replacing the sucrose with sugarcane juice	Addition of sugarcane juice in ice cream increased the total phenolic contents, DPPH free radical scavenging activity, nitric oxide free radical scavenging activity and total antioxidant capacity of ice cream	[124]
Interesterified blends of butter oil and *Moringa oleifera* oil were characterized for antioxidant capacity and storage stability	Phenolic compounds of *Moringa oleifera* oil enhanced the antioxidant perspectives and storage stability of butter oil in long term storage	[125]
Impact of supplementation of ethanolic leaf extract of *Moringa oleifera* on storage stability of butter in refrigeration condition was investigated	Leaf extract of Moringa *oleifera* at 600 ppm may be used for reasonable storage stability of butter at refrigeration temperature with acceptable sensory characteristics	[126]
Effect of almond (*Prunis dulcis*) peel extract was determined on antioxidant characteristics of whey butter	Addition of 400 ppm ethanolic extract of almond peel increased the total phenolic contents and DPPH free radical scavenging activity	[127]
Gouda cheese was supplemented with mango (*Mangifera indica* L.) oil to improve the antioxidant characteristics	Supplementation of mango kernel oil increased the total phenolic contents, DPPH free radical scavenging activity, nitric oxide free radical scavenging activity and inhibited the lipid oxidation	[128]
Influence of intereterified *Moringa oleiefera* oil on oxidative stability of ice cream was studied	Addition of interesterified *Moringa oleifera* oil significantly improved the oxidative stability of ice cream	[129]
The main objective of this study was to raise the antioxidant characteristics of cheddar cheese of chia oil. Cheddar was supplemented with chia (*Salvia hispanica* L.) oil from 2.5 to 10%	Supplementation of cheddar cheese with chia oil increased the antioxidant capacity of cheddar cheese	[130]
Antioxidant characteristics of milk were enhanced by *Hypotrigona squamuligera* honey	Fortification of milk with *Hypotrigona squamuligera* honey inhibited 2,2-diphenyl- 1picrylhydrazyl free radicals with lower peroxide value	[131]

## Ripening effect on antioxidant characteristics of cheese

Cheese is one of the major fermented dairy products. Dairy products are an excellent source of high quality protein and milk fat. It is a rich source of fat soluble vitamins and also an important source of minerals such as calcium, phosphorous and concentrated source of energy [66]. A study was performed in traditional Mexican cheese (Cotiaj) to investigate the antioxidant activities of peptides produced during the 6 months ripening period. Peptides were characterized by HPLC and results showed that peptides with antioxidant activity were produced during the ripening period of 6 months. 2,2-diphenyl-1-picrylhydrazyl (DPPH) free radical scavenging activity of six month old cheese was 98% [67]. Cheddar cheese was prepared using lactobacillus para casei as starter culture and changes in antioxidant characteristics of cheddar was monitored for six months. Different antioxidant assays were used as indicators of antioxidant activity and it was noted that DPPH and superoxide free radical scavenging activities of cheese increased as up to four months of ripening. The increase in antioxidant activities was attributed to the production of water soluble peptides. Antioxidant activity and extent of water soluble peptides were strongly correlated [68]. Antioxidant activity of white brined cheese prepared from overheated milk (90 °C, 10 min) was investigated. Antioxidant activity of water soluble and water insoluble fraction of cheese increased during the ripening period and antioxidant activities were correlated with degree of proteolysis [69]. Effect of phytochemicals on antioxidant characteristics of cheese have been summarized in Table 9.

**Table 9:** Effect of phytochemicals on antioxidant characteristics of cheese

Study design	Conclusions	Reference
Green tea catechins were added in full fat cheeses at 250, 500, and 1000 ppm. Cheeses were ripened for 90 days at 8 °C. Total phenolic content and antioxidant activity of the cheeses were determined	The results showed that addition of GTE significantly decreased the pH of whey and curd during cheese manufacture and ripening, however there was no significant effect on moisture, protein, or fat contents. The addition of gate tea extract increased TPC and AA at all concentrations	[69]
Effect of rosemary leaf supplementation on the antioxidant activities and total phenolic content of Pecorino cheese was studied. Three hundred and twenty-four sheep were randomly assigned to two dietary groups. The concentrate of the rosemary supplemented group contained 2.50% dried rosemary leaves	Results showed that rosemary supplementation increased the total phenolic content, also enhanced the antioxidant properties and decreased the lipid oxidation in cheese	[132]
Effect of catechin on total phenolic content and antioxidant properties in low-fat hard cheese was examined over a 90-day ripening period at 8 °C	Total phenolic content and antioxidant activities were increased during the 90-day ripening period	[133]
Low fat Kalari cheese was treated with different concentrations of pine needle extract (0, 2.5 and 5%), aerobically packaged with polyethylene pouches and kept at 4 °C	Lipid oxidative stability of treated cheese was improved	[134]
The effect of oregano and rosemary essential oils on the oxidative and stability of cream cheese. Peroxide and anisidine values of treated cheese were determined	Supplementation of cream cheese with essentials oil improved the oxidative stability	[135]
Impact of rosemary extract (1.5%) on antioxidant characteristics of soft cheese was studied	Rosemary extract enhanced the antioxidant characteristics of soft cheese	[136]
Extract of fennel (*Foeniculum vulgare*) on antioxidant capacity of cottage cheese was studied	Addition of fennel extract enhanced the shelf life of cottage cheese	[137]
Soft cheeses were supplemented with bay, cinnamon, clove and thyme oils	Phenolic compounds of bay, cinnamon, clove and thyme oils inhibited the *Listeria monocytogenes* and Salmonella in soft cheeses	[138]
Impact of *Matricaria recutita* extract on antioxidant activity of cottage cheese was examined	Cottage cheese functionalized with chamomile extract showed the higher value of antioxidant activity for seven days	[139]
The study was conducted to check the antimicrobial effect of phenolic compounds of *Moringa oleifera* leaf extract in West African soft cheese at 1, 2 and 3% concentration	Phenolic compounds of *Moringa oleifera* lea extact efficiently inhibited the undesirable bacteria in West African Cheese	[140]

## Antioxidant characteristics of yoghurt

The effect of *Pediococcus pentosaceus* on antioxidant characteristics of probiotic yoghurt was studied in cow, goat and camel milk and results evidence that antioxidant of goat milk yoghurt was 93% as compared to 86% in camel milk. These results suggested that antioxidant characteristics of yogurt can be enhanced by probiotic bacteria [70]. Yoghurt is a fermented milk product with distinctive therapeutic value and presented in diversified forms and flavors. Yoghurt was added with carrots, pumpkin, broccoli and red sweet pepper at 10% concentration and ferric reducing antioxidant power (FRAP) and DPPH assays were used for antioxidant activity during the storage period of 14 days. Yoghurt added with broccoli and red sweet pepper revealed higher DPPH free radical scavenging activity and FRAP. However, antioxidant activity decreased during the storage period of 14 days [71]. Similarly, cow, buffalo and goat milk yoghurts were supplemented with aqueous extracts of *Zingiber officinale* and *Beta vulgaris* DPPH free radical scavenging activity and FRAP of goat milk yoghurt was greater than other cow and buffalo milk [72]. In another study, antioxidant capacity of yoghurt was increased by supplementing the yoghurt milk 60 mg vitamin C, 12 mg vitamin E and 3 mg beta-carotene. Antioxidant characteristics of supplemented yoghurts were higher than non-supplemented yoghurts with no effects on sensory properties [73]. Yoghurt was supplemented with fruit pulp of papaya and cactus pear using *Lactobacillus bulgaricus* and *Streptococcus thermphillus* as starter cultures and total phenolic contents, ascorbic acid and total antioxidant activity were analyzed. Yoghurt added with papaya fruit pulp had higher total phenolic contents, antioxidant activity and vitamin C concentration [74]. Typical yoghurt starter bacteria *Lactobacillus delbrueckii* ssp. *bulgaricus* and *Streptococcus thermophiles* inhibited the lipid peroxidation through the scavenging of reactive oxygen species such as hydrogen perode and hydroxyl radicals [75]. Antioxidant activity of milk fermented with *Lactobacillus fermentum* ME-3 was significantly increased higher than milk [76]. The antioxidant activity in fermented dairy products is mainly due to the bioactive peptides released from α-lactalbumin, β-lactoglobulin and α-casein [77].

### In-vivo studies

In-vivo study was performed to assess the antioxidant capacity of the peptides produced in a fermented dairy product similar to yoghurt by the proteolytic strains of *Lb. bulgaricus*. Results revealed that reactive oxygen speices decreased in the live yeast cells [78]. In another in-vivo study, therapeutic perspectives of camel milk were established [79]. The effect of fermented dairy products was observed on the antioxidant enzymes in liver of Swiss mice. Feeding fermented dairy product considerably increased the level of Catalase, Superoxide dismutase (SOD), Glutathione peroxidase and superoxide dismutase [80]. The effect of antioxidant peptides of cow and buffalo cheddar cheese was evaluated against tert-butylhydroperoxide-induced colon cancer. Antioxidant peptides of cow and buffalo milk can protect intestinal epithelium from oxidative damage [81].

### Therapeutic Perspectives of Milk and Dairy Products

Cultured milk has higher antioxidant properties as compared to the normal milk and intake of two servings of cultured milk on daily basis reduced the risk of bladder cancer up to 38% as compared to the people who do not use cultured milk [82]. Intake of milk fermented with *E. faecium* RM11 and *L. fermentum* RM28 had 21 and 29% less chances of colon cancer [83]. In another investigation, it was observed that men and women consuming milk on daily basis had 53% lower risk of bladder cancer while Swedish women containing four servings of high fat milk and dairy products showed 13% less risk of colorectal cancer [84]. Reyes et al. [85] found that milk fermented with *Lactobacillus* spp. and *Bifidobacterium* spp. had a protective effect against liver cancer. Consuming more than 500 ml milk/day significantly decreased the risk of colorectal cancer [86]. Individuals consuming lower amount of milk had higher chances of colorectal cancer [87]. Intake of half liter milk and ricotta cheese on daily basis reduced the risk of colon cancer to 12 and 17% [88]. Intake of 25 g white cheese on daily basis decreased the chances of premenopausal cancer up to 50% as compared to the women consuming less than 6 g white cheese on daily basis [89]. Milk proteins and peptides have shown anti-carcinogenic properties [90]. For instance, lactoferrin is well known for anti-cell proliferation, antioxidant and anti-inflammatory activities [91]. Oral administration of lactoferrin derived from bovine milk considerably reduced the risk of several types of cancers [92]. Casein and whey proteins may protect from colon, breast and prostate gland cancer [93]. The anticancer capability of casein and whey protein may be attributed to the presence of higher concentration of glutathione, which is well known for its antioxidant activity [91]. Immunoglobulins such as IgG1, IgM, IgA and IgG2 has antimicrobial and glutathione enhancing activities which is the important antioxidant of the cell [94]. The association between the dietary intake of milk and dairy products was evaluated and no association was found between the intake of dairy products and cardiovascular diseases [95]. An inverse relation was found between the intake of dairy products and non-fatal cardiovascular disease [96]. No significant association was recorded between the intake of cheese and cardiovascular disease [97]. Intake of low fat, medium fat and high fat cheese had no correlation with cardiovascular disease [98]. Intake of 54 g whey protein on daily basis for a period of 12 weeks significantly reduced the systolic and diastolic blood pressure [99]. Xu et al. [100] observed as strong correlation in the concentration of bioactive peptides produced by the activities of microbiota and gastrointestinal enzymes which are abundantly present in fermented dairy products [101]. A study was conducted on 3435 Parisians for the duration of three days, it was found that higher intake of dairy products led to a lower risk of types 2 diabetes up to 14% [102]. Components of milk and dairy products are industrially used to increase the functional value of the foods. For example, phosphopeptides of casein are used as supplement for several dietary and pharmaceutical applications [103].

## Conclusions

Milk and dairy products, which are basic foods for human development, can be beneficial for the oxidative defence of consumers by several mechanisms. Milk and dairy products with protective properties have the potential to act as coadjuvants in conventional therapies, addressing cardiovascular diseases, metabolic disorders, intestinal health and chemopreventive properties.

## Abbreviations

DNA: Deoxyribo Nucleic Acid
DPPH: 2,2-Diphenyl-1-Picrylhydrazyl
FRAP: Ferric Reducing Antioxidant Power
GSHPx: Glutathione Peroxidase
HPLC: High Performance Liquid Chromatography
SOD: Super Oxide Dismutase
TBA: Thiobarbituric Acid
TPC: Total Protein Content; GTE: Green Tea Catechins
